# Procalcitonin beyond the acute phase: novel biomediator properties?

**DOI:** 10.1186/1741-7015-11-189

**Published:** 2013-08-28

**Authors:** Carolina Panico, Eric Nylen

**Affiliations:** 1 Department Endocrinology, VAMC, and George Washington University School of Medicine, Washington, DC, USA

**Keywords:** Biomarker, Carcinogenesis, Mortality, Procalcitonin

## Abstract

Since inflammation has been linked to carcinogenic events, discovery of relevant biomarkers may have important preventative implications. Procalcitonin (ProCT) has been shown to be an important prognostic biomarker in severe inflammatory conditions, but there is no data regarding its biomarker role, if any, beyond the acute phase. In a recent study published in *BMC Medicine*, Cotoi *et al*. analyzed whether serum ProCT levels in healthy individuals are associated with mortality outcomes. The results are affirmative in that baseline ProCT was shown to be strongly and independently associated with all-cause and cancer mortality and with the incidence of colon cancer in men. By contrast, the study indicated that high sensitivity C-reactive protein was independently associated with cardiovascular mortality but not with cancer mortality in men. Thus, baseline levels of ProCT appear to have prognostic biomarker implications potentially related to its emerging biomediator action(s).

## Procalcitonin

Procalcitonin (ProCT) is the prohormone of the peptide calcitonin normally produced by C cells of the thyroid gland and by certain neuroendocrine cells in the lung. However, in response to bacterial and/or severe systemic inflammatory exposure, cells throughout the body secrete ProCT and its associated peptides (Table 1) [1]. In addition to this biomarker role in septic-like conditions, ProCT also appears to act as a biomediator, whereby it has direct and independent toxic effects on cells [2,3]. Although there has been ample documentation that serum ProCT increases multifold in various septic-like conditions, where it correlates with severity and mortality [1,4,5], there has been little attention drawn to the possible influence of baseline ProCT and its associated peptides that circulate at low concentrations in healthy individuals [6].

**Table 1:** **The ratio of baseline procalcitonin and its associated peptides in the serum of healthy individuals **[6]

**Peptides**	**Ratio**
Calcitonin	1
Procalcitonin	0.1
Aminoprocalcitonin	2.3
Calcitonin carboxyl-terminus peptide-1	1.7

## The Malmo Diet and Cancer Cohort

A recent study published in *BMC Medicine* by Cotoi *et al*. observed the effects of ProCT on all-cause and cancer mortality in healthy individuals [7]. The Malmo Diet and Cancer Cohort is a large longitudinal prospective population study initiated to determine dietary habits and genetic markers that could predict the incidence of cancers in the general population, and to investigate cardiovascular risk factors and early atherosclerosis using three national registries with validated outcomes. Of the 28,449 individuals enrolled in the Malmo Diet and Cancer Cohort, 6,094 were part of the cardiovascular arm of the study. Individuals previously diagnosed with coronary disease or stroke and individuals with previous diagnoses of cancer were excluded from the study. Plasma measurements for ProCT were available for 3,322 participants. In a previous study by these investigators using the same cohort, there was a positive association between plasma levels of ProCT and the incidence of coronary events and deaths in healthy individuals [8]. However, following appropriate statistical adjustments, ProCT was not an independent predictor of cardiovascular risk. Although ProCT is a recognized biomarker, it’s scope of utility has expanded in the recent past to include aspects beyond the acute phase itself. The current study by Cotoi *et al*. recognized that the baseline levels of ProCT might have prognostic features possibly different from those of other biomarkers, such as C-reactive protein [7].

## Procalcitonin and cancer mortality

In the study by Cotoi *et al*., adjusted baseline ProCT was strongly associated with the risk of all-cause and cancer mortality and with incidence of colon cancer in men [7]. The cumulative incidence of all-cause mortality increased by quartile of baseline ProCT levels. By contrast, high sensitivity C-reactive protein was independently associated with cardiovascular mortality but not with cancer mortality in men. Neither biomarker correlated with incident mortality in women. A significant correlation was, however, found between ProCT quartiles and cystatin C, confirming previous data showing that renal function influences plasma levels of ProCT [6]. Although these results are of considerable interest, one important caveat is the assay applied. The assay used for ProCT measurements in the study has its lower detection limit at 10 pg/mL (ProCa-S; BRAHMS GmbH, Hennigsdorf, Germany). This is considered a sensitive assay, but the levels of baseline ProCT obtained, especially in women, are at or below the functional performance of the assay, weakening the overall reliability. As shown in Table 1, ProCT consists of additional peptides other than the intact prohormone itself. For example, the baseline levels of the amino portion of ProCT, which is not detected by the authors’ assay, exceed that of calcitonin and the other peptides, and may therefore yield more accurate information [6].

## Potential pathological pathways

**Figure 1:**
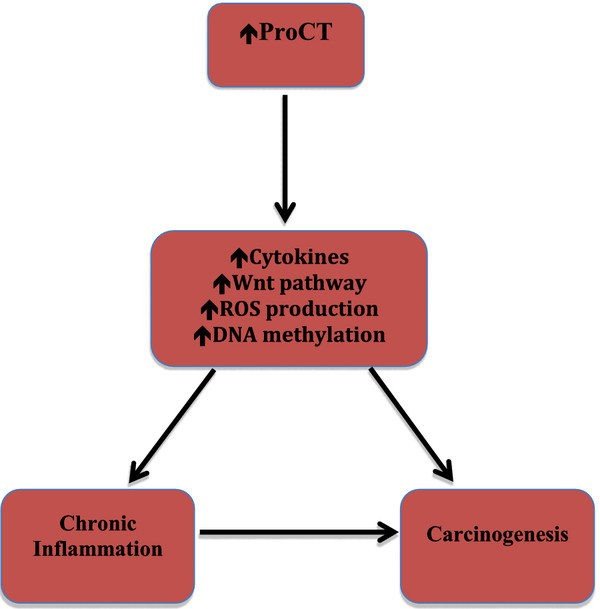
Hypothetical mechanism(s) through which procalcitonin might work as a toxic biomediator.

Although the underlying mechanisms linking ProCT with carcinogenesis are unclear, in the context of the study by Cotoi *et al*. [7], there are several pathological pathways that may have relevance with respect to ProCT (Figure 1).

### Procalcitonin and cytokines

The specific stimuli for ProCT secretion are not well understood although proinflammatory cytokines such as tumor necrosis factor, and IL-1beta have been reported to stimulate ProCT secretion [1]. Moreover, it is known that ProCT induces upregulation of inflammatory cytokines in *in vitro* and *in vivo* studies [2,3,9]. Some of these cytokines are an important link between inflammation and carcinogenesis [10-15]. In particular, IL-6 seems to positively correlate to ProCT levels in various pathological conditions [1,2]. Elevated expression of IL-6 has been detected in various epithelial tumors [16]. Furthermore, several studies implicate IL-6 as a critical mediator of mammary stem cell renewal in both normal and tumor contexts [17].

### Procalcitonin and reactive oxygen species

Various stimuli lead to generation of reactive oxygen species. For example, inducible nitric oxide synthase is associated with cytotoxicity via formation of iron-nitrosyl complexes and inactivation of iron-containing enzymes [18] and may play a role in tumor growth and metastasis [19,20]. Araujo *et al*. observed that inducible nitric oxide synthase is upregulated after the application of ProCT in mesangial cells [2], suggesting that increases in ProCT can be linked to an increase in reactive oxygen species, which in turn has been associated with cancers such as colon cancer [21].

### Procalcitonin and the Wnt pathway

The Wnt pathway has emerged as a critical pathway in several aspects of carcinogenesis [22-24], including aberrant activation in colorectal cancer [25]. Interestingly, the Wnt pathway is significantly upregulated by the application of ProCT [2], suggesting another route for ProCT to carcinogenesis.

### Procalcitonin and DNA methylation

Alterations in circulating DNA can be found in patients with various malignancies, and methylation patterns of serum DNA seem to correlate with clinical presentation and outcome in patients with cancer [15,26]. Interestingly, ProCT correlates with DNA methylation not only in patients with cancer but also those with chronic kidney disease [27].

## Discussion

The results of the study by Cotoi *et al*. highlight novel aspects of ProCT beyond the well-documented acute phase response [1,7]. The fact that ProCT presented a different association pattern from high sensitivity C-reactive protein suggests that ProCT is a biomarker of specific inflammatory processes. Moreover, in the present study, the authors show that after adjustment for cystatin C the association between ProCT and colon cancer in men remained statistically significant, suggesting a specific relationship between cancer and ProCT levels in spite of renal function. Further work is required to determine the potential pathways through which this effect may occur. The importance of the study by Cotoi *et al*. is to uncover a potentially relevant biomarker for early events in the carcinogenic process and thereby enhance preventative strategies. However, essentially nothing is currently known regarding baseline physiological and pathophysiological variability in ProCT or its associated peptides. Most likely, further optimization of the ‘ProCT’ assay would have to be achieved, as discussed above, to establish if the results of Cotoi *et al*. could be used clinically. Intriguingly, ProCT may have a role as a biomediator considering its actions relevant to carcinogenesis (Figure 1); future research may also focus on means to alter ProCT action [28].

## Conclusion

Cotoi *et al*. found an independent link between higher baseline plasma ProCT levels in healthy men with subsequent mortality outcomes and colon cancer, suggesting that subtle but subclinically elevated ProCT might have prognostic implications. Although an epiphenomenon cannot be excluded or causality be proven, the link could be related to emerging biomediator properties of ProCT that merit further scrutiny.

## Abbreviations

IL: Interleukin; ProCT: Procalcitonin.

## Competing interests

The authors declare that they have no competing interests.

## Authors’ contributions

CP reviewed the literature and conceived and produced the illustrations. EN wrote and edited the commentary. Both authors read and approved the final manuscript.
